# Influence of Er, Cr and Diode-980nm lasers on the microhardness of root dentin after cariogenic challenge

**DOI:** 10.4317/jced.62412

**Published:** 2025-03-01

**Authors:** Bruna Alves Castro de Menezes, Larissa Rocha Pacheco, Fernanda Rodrigues Borges Amaral Guarato, Gabriella Rodovalho Paiva, Juliana Jendiroba Faraoni, Regina Guenka Palma-Dibb, Maria Angélica Hueb de Menezes Oliveira, Denise Tornavoi de Castro, Vinícius Rangel Geraldo-Martins, Cesar Penazzo Lepri

**Affiliations:** 1 Department Master’s Program of Dentistry, School of Dentistry of Uberaba, Uberaba, Minas Gerais, Brazil

## Abstract

**Background:**

Root caries is an oral health condition characterized by the demineralization of the root surfaces of teeth. It is particularly common among elderly populations. Lasers have proven to be quite effective in treating root caries, with varying effects depending on the laser parameters and the target tissue. Thus, aim of this article was to evaluate the influence of Er,Cr laser irradiation and 980 nm diode laser application, with or without fluoride treatment, on the microhardness of root dentin.

**Material and Methods:**

Eigthy-bovine incisors were selected (4mm x 4mm x 3mm-thickness) and divided into 8 groups (n=10). NT: no treatment (negative control); FG: Fluoride gel (2%NaF); Er,Cr: Er,Cr: YSGG; Er,Cr+FG; FG+ Er,Cr; Di: 980 nm diode laser; Di+FG; FG+Di. The parameters Er,Cr:YSGG laser were as follows: 0.25 W; 5.0 Hz; 4.46 J/cm2 without water and 55% air. Furthermore, the 980-nm diode laser parameters were 2.0 W; 2.0 Hz; 21.41 J/cm2. Half of each specimen was isolated (control area) and the other half received one of the treatments proposed after to pH cycling during for 15 days to simulate a high caries risk environment, followed by longitudinal microhardness analysis (Knoop) under a load of 30 gf for 30 seconds. Microhardness percentage values were analyzed using the non-parametric Kruskal-Wallis test, followed by Dunn’s post hoc test for pairwise comparisons.

**Results:**

The highest percentage of microhardness loss was observed at 30 μm in FG (35.7%) and in CO (51.9%) (*p*< 0.05).

**Conclusions:**

This study suggests that laser irradiation, with or without fluoride application, is associated with reduced microhardness loss in root dentin.

** Key words:**Dentin, Fluoride, Hardness Tests, Laser, Root caries.

## Introduction

Root caries is an oral health condition characterized by the demineralization of the root surfaces of teeth. It is particularly common among elderly populations, with various epidemiological studies indicating a considerable prevalence (1,2). This phenomenon is attributed to increased longevity, coupled with advancements in dentistry—especially in prevention, diagnosis, and treatment—that allow teeth to remain in the oral cavity for longer periods. While this represents a significant improvement in oral health for the elderly, it also poses challenges for controlling and managing disease progression (3).

With aging, our bodies undergo a series of physiological changes, one of which is bone resorption. This process can lead to the exposure of the root surfaces of teeth (4). When this exposure occurs, teeth become more susceptible to the development of caries. This increased vulnerability is due to the chemical and morphological composition of the root surface, which is more fragile than the dental crown because it lacks enamel. Instead, it is covered only by cementum, which is less resistant (5). Additionally, other age-related factors, such as reduced salivary flow due to medication use or radiotherapy, can further contribute to the development of root caries (6).

An essential biological factor in the formation of root caries lesions is the dental biofilm, although it is not sufficient on its own for these lesions to occur (7). The constant availability of fermentable carbohydrates leads to acid production, promoting microbial adaptations within the biofilm and the proliferation of cariogenic microorganisms. This microbial imbalance results in the re-demineralization of the tooth, as the acidogenic environment favors the growth of acidogenic microorganisms (8). Initially, this manifests as white spots (non-cavitated lesions). If the demineralization process continues, the carious lesion progresses, causing the breakdown of the superficial layer and resulting in a cavity, known as a cavitated carious lesion (9).

One of the most common preventive strategies is the application of fluorides, which exert their effects in various ways. When present in the biofilm and saliva, fluorides slow down demineralization and promote remineralization. Additionally, fluoride interferes with glycolysis, the process by which cariogenic bacteria convert sugars into acid (10). However, the effect of fluoride is limited, as it does not completely prevent disease development under high cariogenic challenges. Furthermore, root caries lesions involve both demineralization and the degradation of the organic matrix of dentin (11).

Lasers have proven to be quite effective in treating root caries, with varying effects depending on the laser parameters and the target tissue. Key parameters include wavelength, pulse duration, and energy density, all of which influence the effects of lasers on tissues (12,14). Evidence suggests that laser irradiation can enhance the resistance of both dentin and enamel to demineralization, making them less susceptible to caries formation (13-15). Additionally, the application of lasers modifies the structure of the dentin surface, which can reduce the solubility of minerals and increase resistance to mineral loss due to acidic challenges in the oral cavity (16).

When lasers are applied, the temperature of the dental surface increases. This thermal rise decreases the dissolution of hydroxyapatite crystals (17). Incomplete degradation of the organic matrix of enamel during laser irradiation blocks intra- and inter-prismatic spaces, preventing the spread of acids into the inner layers of the enamel and slowing down the demineralization process (17). In dentin, the application of high energy densities has been shown to inhibit demineralization by stimulating the formation of recrystallization zones, as well as the fusion and melting of the tissue (13). The use of lasers has the potential to induce dentin recrystallization, resulting in dentin with a higher degree of crystallinity. This structural modification causes dentin to resemble the crystalline structure of hydroxyapatite found in healthy enamel (18).

The Er,Cr laser has demonstrated effectiveness in some studies due to its high absorption at the wavelength of 2.78 μm in water and hydroxyapatite, which are key components of dental tissue. Irradiation with the erbium laser promotes the selective removal of water and deproteinization of dentin, facilitating recrystallization and increasing the size of hydroxyapatite crystals (19).

In contrast, diode lasers offer several significant advantages. Firstly, their compact size allows for easy integration into dental office without requiring major adjustments to the physical space. Additionally, compared to other types of dental lasers, diodes are relatively inexpensive, making them an accessible option for many dental professionals (20). The mechanism of action of diode lasers primarily occurs through a photothermal process. When applied to the surface of the dental root, the heat generated can eliminate existing bacteria, thereby reducing the risk of root caries. This mechanism also stimulates mineral deposition, promoting the remineralization of demineralized areas. These characteristics provide a less invasive and potentially more effective approach compared to traditional methods (21).

Given the increasing prevalence of root caries, it is essential to evaluate the efficacy of Er, Cr laser, and 980 nm diode laser, both alone and in combination with fluorides, compared to conventional methods. This study aims to elucidate the effects of these treatments on root dentin microhardness, providing robust information to improve therapeutic and clinical strategies for the prevention and control of caries disease.

A detailed analysis of these methods may offer new perspectives for developing more effective and less invasive approaches to managing root caries. The hypothesis of the present study was that preventive treatments with Er, Cr: YSGG, and 980nm Diode laser irradiation associated or not with fluoride would result in significant differences in the longitudinal microhardness of bovine root dentin under cariogenic conditions.

## Material and Methods

1. Experimental design

The factors under study were the preventive treatment methods (2% fluoride gel, Er,Cr:YSGG laser irradiation, 980 nm diode laser irradiation and 2% fluoride gel combined with laser) and the cariogenic challenge. According to the preventive treatment performed, the experiment sample had 80 test specimens divided into 8 groups.

This study used a randomized complete block design, with each experimental group repeated per block. The quantitative response variable was the longitudinal microhardness of the dentin (in %).

2. Selection of teeth

After receiving approval from the ethics committee for animal experimentation at the University of Uberaba (protocol 008/2023), eigthy bovine central incisors were selected for this study (22). The bovine teeth were cleaned using periodontal curettes, and any remaining debris was removed with a Moto Esmeril (Tramontina 6” bivolt 368W) equipped with a circular steel brush (0.3mm wire), ensuring the complete removal of the cementum layer. The teeth were then thoroughly rinsed and stored in distilled water at 4°C, with the water being changed weekly (15).

3. Preparation of specimens

The dental roots were carefully separated from the crowns 1 mm from the enamel-cement junction using a diamond disc with water cooling, attached to a cutting machine. A second cut was made with a precision cutting machine, sectioning the roots in a cervical-apical direction, resulting in specimens measuring 4.5 mm. A third cut on the precision machine produced two halves: one mesial and one distal. Each specimen was then polished on a metallographic polisher (APL) using #360 water sandpaper, resulting in standardized blocks measuring 4.25 mm x 4.25 mm x 3.00 mm thick, with a surface area of approximately 18.0 mm² (9 mm² for the experimental area and 9 mm² for the control area). A variation of ±5% in 

To identify the experimental and control areas, a hole was drilled with a spherical diamond tip (FG 1013) on the lateral face of the experimental area of all specimens. The experimental face was then covered with insulating tape, while the control area and other faces were sealed with a double layer of red nail polish. After the nail polish dried, a layer of sticky sculpting wax was applied to all faces except the experimental half. The insulating tape was subsequently removed, exposing one dentin face for treatment and irradiation. The specimens were stored in distilled water at 4°C until the preventive treatments were performed.

3. Specimen treatment/experimental groups (n=10)

The specimens were divided into 8 groups the according to the treatment, as follows: NT: no treatment (negative control); FG: Fluoride gel (2%NaF); Er, Cr: Er, Cr: YSGG; Er, Cr+FG; FG+ Er, Cr; Di: 980 nm diode laser; Di+FG and FG+Di.

Application methods for each treatment:

- Fluoride Gel: A 2% neutral fluoride gel was applied to the dentin surface using a microbrush and left for 4 minutes. After this time, the excess gel was removed with gauze 

 -Laser Parameters: The following varying factors were taken into consideration: wavelength, power, emission mode, repetition rate, thickness of the optical fiber, distance between the laser output lens and the target tissue, total irradiation time on the surface of each specimen, and the presence or absence of cooling during irradiation, as described in  Table 1 and  Table 2.

Water cooling was not used to avoid compromising the treatment, as water could cause ablation (13-15). During irradiation, only the irradiated group and the groups that received the Er, Cr laser before the gel were exposed to 55% air. In the groups where irradiation occurred simultaneously with the applied gel, the air/water system was turned off to prevent the removal of the gel fluoride.

4. Cariogenic Challenge (pH Cycling)

The samples from each group were subjected to pH cycling to simulate a situation of extremely high caries risk. Each group, containing the samples, was stored in its respective plastic container, where demineralizing (DES) and remineralizing (RE) solutions were added and replaced. A volume of 50 mL of the demineralizing solution (2 mmol/L calcium, 2 mmol/L phosphate, and 75 mmol/L acetate at pH 4.6) was placed in the corresponding containers, and the samples were immersed for 6 hours.

Afterward, the samples were removed, thoroughly rinsed with distilled water for 10 seconds, and gently dried with gauze. The containers were also washed and dried. Next, a volume of 50 mL of the remineralizing solution (1.5 mmol/L calcium, 0.9 mmol/L phosphate, 150 mmol/L potassium chloride, and 20 mmol/L cacodylate buffer at pH 7.0) was placed in the corresponding containers, and the samples were immersed for 18 hours. The remineralizing solution has a mineral saturation level similar to that of saliva.

The demineralizing (DES) and remineralizing (RE) solutions were replaced daily, and the cycling continued for 15 days. After 5 days, the samples were individually immersed in the remineralizing solution for 2 days (over the weekend), resulting in a total experimental period of 14 days. Throughout this time, the samples were stored in an incubator at 37°C.

5. Analysis of Longitudinal Knoop Microhardness

The specimens were included in polyester resin and sectioned crosswise. After the polishing of these surfaces with silicon carbide (#600 and #1200) and felt disk with alumina, control and experimental area were evaluated, using aload of 30gf by 30s at the following depths: 30μm, 60μm and 250 μm (Shimadzu HMV 2000, Shimadzu Corporation Kyoto, Japan). For each depth, three measurements were taken, with an approximate distance of 500 μm between them.

 6. Statistical Analysis

In the present study, the sample size was calculated considering a significance level of 5% and the power of the statistical test of at least 85%, thus minimizing the chances of type I and type II errors, respectively.

The percentage values of microhardness (%) were subjected to the non-parametric Kruskal-Wallis statistical test, followed by Dunn’s post-test.

## Results

**Figure 1:**
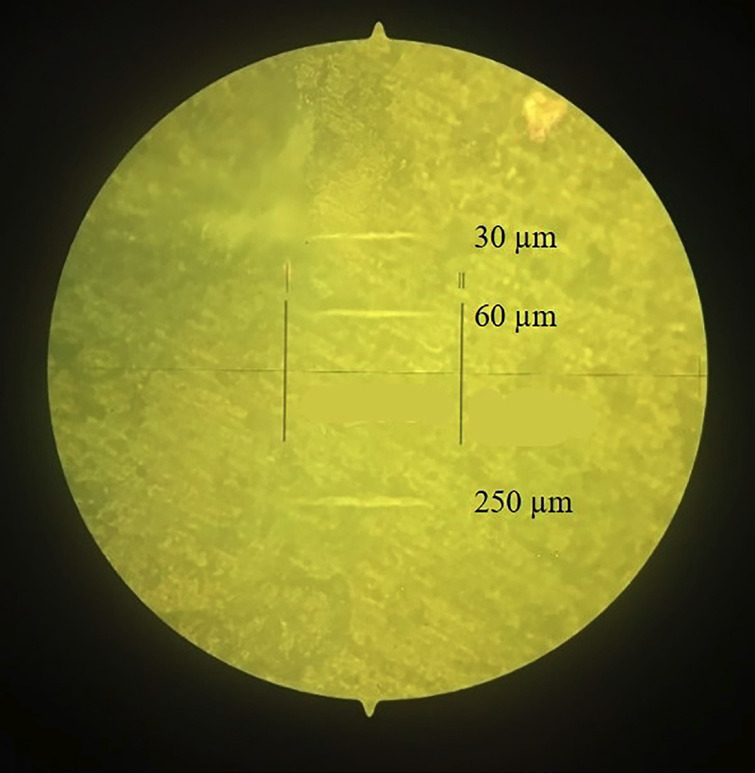
Illustrative image of dentin tissue after microhardness analysis, at depths of 30 µm,60 µm,250 µm.

The data obtained for microhardness are described in  Table 3. The penetrations carried out to analyze microhardness at depths of 30 µm,60 µm and 250 µm are represented in Figure 1.

At a penetration depth of 30 µm, the groups Er, Cr; FG + Er, Cr; Di; Di + FG and FG + Di did not show statistically significant differences among themselves (*p*>0.05) regarding microhardness. In contrast, the FG and NT groups exhibited a greater reduction in microhardness.

At a depth of 60 µm, the groups Er, Cr; Er, Cr + FG; FG + Er, Cr; Di; Di+ FG; FG+ Di; and FG did not demonstrate statistically significant differences among themselves (*p*>0.05) concerning microhardness. However, the NT group showed a greater reduction in microhardness, which was statistically different from the other groups (*p*<0.05).

At a depth of 250 µm, all groups did not exhibit statistically significant differences among themselves (*p*>0.05).

## Discussion

Caries prevention, especially on root dentin surfaces, has become a research focus due to its increasing prevalence in elderly populations. Therefore, it is crucial to evaluate the efficacy of Er, Cr laser, and 980 nm diode laser, individually and in combination with fluoride, compared to conventional methods. A detailed analysis of these methods may offer new insights for developing more effective and less invasive approaches for managing root caries. The hypothesisof this study was confirmed, as the results indicated a significant difference in preventive efficacy against caries between the use of lasers alone and combination with fluoride.

The results of this study suggest that laser treatments, both the 980 nm diode and Er,Cr lasers, whether used in conjunction with fluoride gel or not, were effective in reducing the loss of microhardness of root dentin at all analyzed depths (30 µm, 60 µm, 250 µm), except for the isolated application of fluoride gel, which showed significant differences at a depth of 30 µm. At a depth of 60 µm, only the untreated group exhibited an increase in the loss of microhardness. At the 250 µm penetration depth, there were no statistical differences between the groups (*p* > 0.05). This variation in results at different depths occurs because the acid does not penetrate deeply, resulting in greater demineralization at the 30 µm level.

The choice bovine teeth was made due to their greater standardization. They were based on previous studies in which tests were performed that demonstrated that human and bovine dentin substrates had similar morphology (22), an was obtained from elemental analysis that human enamel and dentin were more similar to bovine teeth. Therefore, based on their chemical composition, bovine teeth should be the first choice as a substitute for human teeth in research.

However, in another study (23), a different result was observed: the surface hardness of dentin significantly decreased after the application of Er, Cr and Er lasers, showing significant differences between the experimental groups. The subsequent cariogenic challenge performed in that study likely intensified the decrease in microhardness. Furthermore, the parameters used for the Er, Cr laser were 3.5 W of power with 70% water and 60% air, unlike the present study, which utilized 0.24 W of power and no water cooling to avoid compromising the preventive treatment, as the presence of water can cause ablation of the dentin tissue (13-15).

Moreover, a study by Geraldo-Martins *et al*. (2014) demonstrated that the association of Er, Cr laser and fluoride can increase the acid resistance of human root dentin when irradiation is performed without water cooling (24). Samples irradiated with 0.25 W and 0.50 W without water cooling, followed by the application of 2% NaF, showed surfaces that were more resistant to acids. This effect was observed at depths of 30 and 60 μm, with no statistically significant differences at depths of 90 and 120 μm, similar to the present study. The lack of water cooling may lead to an increase in surface temperature, promoting effects similar to those in enamel, where elevated temperatures cause changes in the chemical structure, making it a less soluble material (25).

Furthermore, similar results to those of this study were documented. Lepri *et al*. (2022) found that the Er laser can safely increase the acid resistance of human root dentin surfaces (14). This significant increase in surface hardness occurs without causing charring or cracks in the dental structure, indicating that erbium laser treatment is effective in caries prevention and improves the integrity of root dentin. Additionally, the combination of 2% NaF application with laser irradiation did not show a synergistic effect on increasing acid resistance. This is consistent with the present study, which found no statistical differences between the application of the laser alone and its use in conjunction with fluoride.

Regarding the 980 nm diode laser, it was found that irradiation with this type of laser can modify the chemical composition of dentin, increasing its resistance to acid demineralization. When applied to the root dental surface, a photothermal effect occurs; the heat generated by the laser can eliminate existing bacteria, significantly reducing the risk of root caries. Moreover, it promotes mineral deposition, facilitating the remineralization of demineralized areas. These characteristics make laser treatment a less invasive and potentially more effective approach compared to traditional methods (21).

In a study involving the 980 nm diode laser, it was suggested that the laser may form a less acid-soluble layer of dentin, contributing to protection against caries (26). Furthermore, research conducted by Viapiana *et al*. (2012) revealed that the microhardness of groups irradiated with the 980 nm diode laser was significantly greater compared to the non-irradiated group, particularly at depths of 150 µm and 300 µm (27). This contrasts with the present study, which showed more satisfactory results at depths of 30 and 60 µm. This discrepancy likely occurred due to the short duration of the acid challenge, which did not allow the demineralizing solution to penetrate the deeper layers of root dentin (90 and 250 μm). However, these results indicate that the 980 nm diode laser can strengthen the resistance of root dentin, making it less susceptible to demineralization and reinforcing the dental structure.

Regarding fluoride gel, the study by Byeon, Lee, and Bae (2016) demonstrated that fluoride gel showed some benefit when combined with other fluoride sources in dentin (28). However, it was noted that a higher concentration of fluoride was needed to inhibit demineralization and promote remineralization compared to enamel.

In the present study, the use of fluoride gel was effective in protecting dentin tissue; however, the best results were achieved when combined with laser treatment or when the laser was applied alone. This effectiveness is attributed to the laser’s effect; the application of high energy densities stimulates the formation of recrystallization zones, melting, and fusion of the tissue (13), leading to a more effective incorporation of fluoride into dental tissue.

Vale *et al*. (2010) demonstrated that the use of fluoride gel alone is insufficient to protect the root surface against the attack of acidogenic bactéria (29). Acidogenic bacteria are microorganisms that produce acids as a byproduct of fermenTable carbohydrate metabolism, leading to the demineralization of dental tissue. Fluoride in gel form may not adequately neutralize these acids or strengthen the structure of root dentin, leaving it vulnerable to dental caries. Indeed, the results of the present study indicated a greater percentage of microhardness loss with the application of fluoride gel alone.

A study involving the use of lasers in combination with fluorides also yielded promising results. One research found that the combination of fluoride varnish with the 980nm diode laser and the Er, Cr laser resulted in the lowest values of surface roughness and volume loss compared to other analyzed groups (17). This demonstrates that the synergism between the laser and fluoride can significantly enhance the acid resistance of human dentin, promoting morphological changes in the dentin structure, making it more similar to enamel, less soluble, and more resistant to acid challenges.

These findings reinforce the importance of using lasers as effective tools in caries prevention, especially on root dentin surfaces. The ability of the 980 nm diode and Er, Cr lasers, whether used alone or in combination with fluoride, to reduce the percentage of microhardness loss in dentin suggests that they can be effectively integrated into preventive protocols, offering a less invasive and potentially more effective alternative to traditional methods.

The limitations of this study were: lack of different situations that teeth are subjected to in the oral cavity, such as changes in temperature and pH, factors related to saliva and ion release between dental substrates, in addition to the absence of microorganisms and use of bovine tooth (10,11,15,22).

Further studies are needed to evaluate physical-mechanical properties such as adhesion to the irradiated substrate, surface morphology of dentin, analysis of decayed tissue by microorganisms, and alternative parameters of the lasers used in this study, as well as clinical studies and long-term effects.

In the face of the results found in this study, the clinicians should advise his patients on the importance of existing preventive methods for caries and their benefits, given that demineralization promotes the reduction of tooth hardness or that it is prejudicial to the maintenance of dental structures and of the non-general odontological procedures.

## Conclusions

Considering the limitations of an *in vitro* study, this research demonstrated the efficacy of irradiation with Er, Cr laser, and 980 nm diode laser, used or not in association with fluoride application, in reducing the percentage of microhardness loss in root dentin. However, clinical studies are needed to evaluate the treatments tested and investigate alternative laser protocols for promising findings.

## Figures and Tables

**Table 1:** Irradiation parameters of the Er, Cr: YSGG laser.

Irradiation Parameters	Er, Cr:YSGG
Power	0,25W
Repetition Rate	5,0Hz
Energy Density	4,46J/cm²
Irradiation time	10 seconds
Mode	Surface Scanning
Target Distance	1 mm from target tissue
Air/Water	No water cooling

**Table 2:** Irradiation parameters of the Diode laser -980nm.

Irradiation Parameters	Diode-980nm
Power	2,0W
Repetion rate	2,0Hz
Energy Density	21,41J/cm²
Irradiation time	10 seconds
Mode	Surface scanning
Target Distance	Direct contact with target tissue
Air/Water	No water cooling and no air

**Table 3:** Percentage reduction of microhardness in the groups, Comparing the Experimental Area (EA) and the Control Area (CA) within each group. ((EA-CA)/CA).

Groups	Treatments	30 µm	60 µm	250 µm
NT	No treatment	51,9^c^	20,8^b^	3,1^a^
FG	Fluoride Gel	35,7^b^	10,2^a^	2,5^a^
Er,Cr	Er,Cr:YSGG Laser	15,1^a^	8,6^a^	2,1^a^
Er,Cr+FG	Er,Cr + Fluoride Gel	15,6^a^	8,5^a^	2,0^a^
FG+Er,Cr	Fluoride Gel + Er,Cr:YSGG	13,5^a^	7,8^a^	2,0^a^
Di	Diode-980nm	16,0^a^	9,1^a^	2,3^a^
Di+FG	Diode-980nm + Fluoride Gel	15,9^a^	9,3^a^	2,4^a^
FG+Di	Fluoride Gel + Diodo-980nm	14,7^a^	8,8^a^	2,1^a^

Equal letters indicate statistical similarity between groups (*p*>0.05).

## Data Availability

The datasets used and/or analyzed during the current study are available from the corresponding author.

## References

[1] Souza JGS, Oliveira BEC, Sampaio AA, Lages VA, Romão DA, Martins AM (2018). Contextual and individual determinants of root caries in older people. Caries Res.

[2] Reddy L, Lakshmi S, Lakshmi Y, Lakshmi KD, Sravanthi Y, Kaur M (2021). Root caries experience and its association with risk indicators among middle-aged adults. J Pharm Bioallied Sci.

[3] Grandjean ML, Maccarone NR, McKenna G, Müller F, Srinivasan M (2024). Silver diamine fluoride (SDF) in the management of root caries in elders. Swiss Dent J SSO - Sci Clin Topics.

[4] Bahrami G, Vaeth M, Wenzel A, Kirkvang LL, Isidor F (2011;19). Prediction of future marginal bone level: a radiographic study. J Clin Periodontol.

[5] Zhao IS, Gao SS, Hiraishi N, Burrow MF, Duangthip D, Mei ML (2018). Mechanisms of silver diamine fluoride on arresting caries: a literature review. Int Dent J.

[6] Chan AKY, Tamrakar M, Jiang CM, Tsang YC, Leung KCM, Chu CH (2022). Clinical evidence for professionally applied fluoride therapy to prevent and arrest dental caries in older adults: a systematic review. J Dent.

[7] Qutieshat A, Salem A, Aouididi R, Bronzato JD, Al-Waeli H, Abufadalah M (2021). Perspective and practice of root caries management: a multicountry study part II. J Conserv Dent.

[8] Al-Nasser L, Lamster IB (2020). Prevention and management of periodontal diseases and dental caries in older adults. Periodontol 2000.

[9] O'Hagan-Wong K, Enax J, Meyer F, Ganss B (2021). The use of hydroxyapatite toothpaste to prevent dental caries. Odontology.

[10] O'Mullane DM, Jones RJ, Baez S, Lennon MA, Petersen PE, Rugg Gunn AJ (2016). Fluoride and oral health. Community Dent Health.

[11] Cury MS, Silva CB, Nogueira RD, Campos MG, Palma-Dibb RG, Geraldo-Martins VR (2017). Surface roughness and bacterial adhesion on root dentin treated with diode laser and conventional desensitizing agents. Lasers Med Sci.

[12] Moghadam NCZ, Seraj B, Chiniforush N, Ghadimi S (2018). Effects of laser and fluoride on the prevention of enamel demineralization: an in vitro study. J Lasers Med Sci.

[13] Arantes BF, Mendonça LO, Palma-Dibb RG, Faraoni JJ, Castro DT, Geraldo-Martins VR (2019). Influence of Er, Cr laser, associated or not with desensitizing agents, in the prevention of acid erosion in bovine root dentin. Lasers Med Sci.

[14] Lepri CP, Castro DD, Geraldo-Martins VR, Faraoni J, Palma-Dibb RG (2022). Laser irradiation prevents root caries: microhardness and scanning electron microscopy analysis. Indian J Dent Res.

[15] Guarato FRBA, Santi MR, Madalena IR, Geraldo-Martins VR, Oliveira MAHM, de Castro DT (2024). Er, Cr and 980 nm diode lasers influence dentin surface volume after cariogenic challenge: in vitro study. Braz Oral Res.

[16] Valizadeh S, Rahimi Khub M, Chiniforush N, Kharazifard MJ, Hashemikamangar SS (2020). Effect of Laser Irradiance and Fluoride Varnish on Demineralization Around Dental Composite Restorations. J Lasers Med Sci.

[17] Nogueira RD, Silva CB, Lepri CP, Palma-Dibb RG, Geraldo-Martins VR (2017). Evaluation of Surface Roughness and Bacterial Adhesion on Tooth Enamel Irradiated with High Intensity Lasers. Braz Dent J.

[18] Hoshyari N, Zamanian A, Samii A, Mousavi J (2023). In-vitro Comparison of Occluding Effect of Fluoride Varnish and Diode Laser Irradiation with Fluoride Varnish and Er,Cr:YSGG Laser Irradiation on Dentinal Tubules of the Cervical Root Area of the Tooth. Maedica (Bucur).

[19] Maddah F, Shirinzad M, Khalafi Z, Rezaei-Soufi L, Mohammadi Y, Eskandarloo F et al (2023). Synthesis and characterization of hydroxyapatite nanoparticles and their effects on remineralization of demineralized enamel in the presence of Er, Cr laser irradiation. BMC Oral Health.

[20] Abdelkarim-Elafifi H, Parada-Avendaño I, Arnabat-Domínguez J (2022). Parameters used with diode lasers (808-980 nm) in dentin hypersensitivity management: a systematic review. J Lasers Med Sci.

[21] Ghazy B, Fawzy M, Abdelkafy H (2022). Effect of irradiation with 980-nm diode laser on the microhardness of young and old root canal dentin after treatment with chemical solutions. Al-Azhar J Dent.

[22] Teruel Jde D, Alcolea A, Hernández A, Ruiz AJ (2015). Comparison of chemical composition of enamel and dentine in human, bovine, porcine and ovine teeth. Arch Oral Biol.

[23] Al-Omari WM, Palamara JE (2013). The effect of Nd: YAG and Er, Cr: YSGG lasers on the microhardness of human dentin. Lasers Med Sci.

[24] Geraldo-Martins VR, Lepri CP, Faraoni-Romano JJ, Palma-Dibb RG (2014). The combined use of Er, Cr laser and fluoride to prevent root dentin demineralization. J Appl Oral Sci.

[25] Abdulhussein DN, Al Haidar AMJ (2023). Preventive effect of combined Er, Cr: YSGG and fluoride gel on acid resistance of the permanent tooth enamel: An in vitro study. J Clin Exp Dent.

[26] Lopes FC, Roperto R, Akkus A, Akkus O, Souza-Gabriel AE, Sousa-Neto MD (2016). Effects of different lasers on organic/inorganic ratio of radicular dentin. Lasers Med Sci.

[27] Viapiana R, Sousa-Neto MD, Souza-Gabriel AE, Alfredo E, Silva-Sousa Y (2012). Microhardness of radicular dentin treated with 980-nm diode laser and different irrigant solutions. Photomed Laser Surg.

[28] Byeon SM, Lee MH, Bae TS (2016). The effect of different fluoride application methods on the remineralization of initial carious lesions. Restor Dent Endod.

[29] Vale GC, Tabchoury CPM, Cury AAB, Tenuta LMA, Cury JA (2010). APF and dentifrice effect on root dentin demineralization and biofilm. J Dent Res.

